# From Measurement to Analysis Reporting: Grand Challenges in Nutritional Methodology

**DOI:** 10.3389/fnut.2014.00006

**Published:** 2014-06-26

**Authors:** Tapan Mehta, David B. Allison

**Affiliations:** ^1^Department of Physical Therapy, Nutrition Obesity Research Center, University of Alabama at Birmingham, Birmingham, AL, USA; ^2^Department of Biostatistics, Office of Energetics and Nutrition Obesity Research Center, University of Alabama at Birmingham, Birmingham, AL, USA

**Keywords:** observations, measurements, designs, analyses, interpretation

Nutrition has been central to the progress and well-being of mankind. Nutritional treatments and interventions, nutritional public policy and guidelines, issues related to agriculture and the food supply, nutritional genomics for individualized treatments, and other such topics remain vital areas of scientific inquiry. The foundation of progress in science is rigorous methods. Many of our methods have improved substantially over the years, yet there are always healthy calls for ever greater rigor in nutrition research (1–3). Ongoing advancement in methodological aspects of nutrition, energetics, and obesity studies include at least three components: (a) observations and measurements, (b) experimental design, and (c) statistical analysis and interpretation. We challenge ourselves and the field to expand the frontiers of nutritional knowledge by advancing the breadth, the rigor, and the quality of use of scientific methods in nutrition-related research.

The beginnings of modern medicine and nutritional science can be dated to the early seventeenth century, when the old ideas of the Four Humors Theory (4) were finally being questioned. Back then it was customary to attribute any disease to one of the four body humors without careful measurements or analysis. Jean-Baptiste van Helmont was one of the first investigators who chose to take detailed measurements. Incidentally, this was the same era in which Galileo questioned old Greek ideas and developed devices such as the thermometer, which van Helmont used to measure temperatures. Sanctorius further improvised this device to develop a clinical thermometer for examining sick individuals (4). It was also during the seventeenth century when medically oriented scientists focused on developing microscopes, an advancement from the glass lens that had been developed since the thirteenth century. The invention of the microscope was critical to advancing our understanding of general medical science, including nutritional research. Indeed, microscopes were crucial in our understanding the processes of energy production and chemical interaction and in the study of animal fluids (5). Analogously, in modern times, advances like the Google Glass (6) may set the stage for us to develop next-generation data collection methods and protocols.

Our first challenge is to advance data collection methods by using an ensemble of cutting-edge technological advances from fields such as biophysics, bioengineering, psychometrics, nanotechnology, and biomaterials (7–10). Studies to estimate measurement errors and biases in data collection techniques will also aid in advancing the reliability and accuracy of data collection methods (9). This challenge includes reducing some of the problems with or supplanting some outmoded existing data collection methods such as self-reported dietary intake (11, 12).

The maturing of nutritional research from measurements and observations to intervention study design can be dated to James Lind’s scurvy trial in the eighteenth century (13). Lind divided 12 sailors affected with scurvy into 6 pairs and treated each pair with a different nutritional intervention. These 12 sailors were selected such that they were similar in terms of general characteristics such as diet as well as symptoms of scurvy. Lind discovered that the two patients who had lemons and oranges clearly showed the best results. This nutrition trial by Lind can be viewed as the first controlled, but not randomized, comparative clinical trial (4). Later in the early twentieth century, Fisher championed the idea of randomization when he was assigned to study and improve wheat yields and in a series of papers proposed ideas about randomization and blocking with respect to agricultural experimentation (13, 14).

Randomized controlled experiments using animal models or model organisms also provide us with a unique opportunity to understand the biological, behavioral, and other processes in play when evaluating interventions. The value and importance of conducting RCEs in humans including randomized controlled trials (RCTs) in nutrition and obesity research is profound. However, conducting these RCEs in humans for clinically relevant traits for clinically relevant durations can be demanding. As a result the field has relied heavily on observational study designs (cross-sectional or longitudinal) where the individuals are not assigned to levels of independent variables by the experimenter or at random. Findings from these studies are then based on ordinary tests of associations (OATs) where the only or primary means of controlling for potential confounding is by including selected measured variables in the analysis as covariates, matching on selected measured variables, or by restricting the analysis to certain factors. While OATs are useful and necessary at times, in nutrition research, OATs have a poor track record for identifying effects that hold up in RCTs. Therefore, it has been difficult to affirm the clinical or public health value of the findings from OATs in epidemiological studies (15–19). Because of these factors, it is important that, whenever possible, we go beyond sole reliance on OATs from epidemiological studies, even when RCTs are not viable. Evidence of causation exists on a continuum, and a variety of designs are available, such as co-twin control studies, quasi-experiments, and natural experiments, that are intermediate between OATs and RCTs (20). Our next challenge is to be creative and bold in designing studies that utilize the full range of this continuum instead of reflexively settling with OATs. This is especially important given that one of the central underlying themes of research related to nutrition has been to identify causal effects of interventions.

The quality of the statistical analysis, interpretation of the results, and accurate reporting of findings are key aspects of nutritional research. The quality of an analytic approach of a study is influenced by factors such as: (1) validity and performance of the chosen statistical method in realistic settings; (2) intelligent use of the method; (3) availability of software; and (4) fair and transparent reporting and interpretation of results. Validity of the statistical methods applied in any scientific inquiry is a necessary aspect of its sound epistemological foundations and it is important to assess the validity rigorously (21, 22). Simulation and plasmode studies allow assessment of the performance of statistical methods better than ever before (23). Too often, the choice of statistical analysis seems driven by tradition or convention, rather than a thoughtful consideration of available options (24, 25). Novel and valid analytic approaches need to be proposed to address statistical issues to overcome the limitations of existing statistical approaches (26). Unavailability of software that provides valid and specialized statistical approaches can also limit the analytic choices. Part of improving the standards of statistical analyses in nutritional research would also be developing software that disseminates valid statistical methods applicable to the field and supports reproducible research.

Finally, nutrition studies, perhaps especially observational studies, which are widely used in nutritional research, have been subject to publication and reporting biases (27, 28). Hence, a need also exists to improve standards of reporting and interpreting the results, which are sometimes misreported (2, 27, 29). Studies also report varying and sometimes conflicting results, at least in part, due to analytic and design differences (30–32). Rigorous meta-methods, which include systematic reviews, meta-analyses, meta-regression, and pooled analyses of individual-level data, offer opportunities to summarize and integrate the evidence across studies in a valid manner. These meta-method studies, which have beginnings in early 1900s but remained under-utilized in the field of epidemiology, have been gaining popularity (33, 34). Meta-analyses or systematic reviews that rely primarily on the published literature are also subject to publication and search bias (35). A well-designed individual-level pooled data analysis offers more flexibility in synthesizing evidence, when the individual-level data are available (35). Recent advances in explicit guidelines and requirements, to conduct meta-analyses and systematic reviews, also help document and alleviate some of these issues related to synthesizing the evidence (36, 37). However, reporting studies of meta-methods is not consistently met up to these standards prescribed by the guidelines. There remains a need and opportunity to further the role of these meta-methods in reporting the evidence across studies, improving the standards of transparency, and improving methods in conducting meta-analyses (38). Thus, our final grand challenge is to elevate the overall standards of statistical analyses and reporting in nutritional research through: the development of novel methods and intelligent use of existing methods including meta-methods; evaluation of methods in realistic settings; evaluating standards of reporting and interpretation in the literature; and improving the standards of transparency for meta-method studies.

The history of science shows that advances in knowledge frequently preceded and were made possible by advances in methods that allow us to see what we could not see before. We look forward to working with all of you to see further than we have.

## Conflict of Interest Statement

The authors declare that the research was conducted in the absence of any commercial or financial relationships that could be construed as a potential conflict of interest.
